# The endocannabinoid system is involved in the anxiety-like behavior induced by dual-frequency 2.65/0.8 GHz electromagnetic radiation in mice

**DOI:** 10.3389/fnmol.2024.1366855

**Published:** 2024-04-15

**Authors:** Teng Xue, Rui-Han Ma, Chou Xu, Bin Sun, Dong-Fei Yan, Xiao-Man Liu, Dawen Gao, Zhi-Hui Li, Yan Gao, Chang-Zhen Wang

**Affiliations:** ^1^Laboratory of Bioelectromagnetics, Beijing Institute of Radiation and Medicine, Beijing, China; ^2^School of Life Sciences, Hebei University, Baoding, Hebei, China; ^3^Department of Critical Care Medicine, The 983rd Hospital of the Joint Logistics Support Force of PLA, Tianjin, China; ^4^Chinese PLA General Hospital, Beijing, China; ^5^Center of Cognition and Brain Science, Beijing Institute of Basic Medical Sciences, Beijing, China

**Keywords:** 2.65 GHz, 0.8 GHz, electromagnetic radiation, anxiety-like behavior, endocannabinoid system, cerebral cortex

## Abstract

As wireless communication devices gain popularity, concerns about the potential risks of environmental exposure to complex frequency electromagnetic radiation (EMR) on mental health have become a public health issue. Historically, EMR research has predominantly focused on single- frequency electromagnetic waves, neglecting the study of multi-frequency electromagnetic waves, which more accurately represent everyday life. To address these concerns, our study compared the emotional effects of single-frequency and dual-frequency EMR while exploring potential molecular mechanisms and intervention targets. Our results revealed that single-frequency EMR at 2.65 or 0.8 GHz did not induce anxiety-like behavior in mice. However, exposure to dual-frequency EMR at 2.65/0.8 GHz significantly led to anxiety-like behavior in mice. Further analysis of mouse sera revealed substantial increases in corticosterone and corticotrophin releasing hormone levels following exposure to 2.65/0.8 GHz EMR. Transcriptome sequencing indicated a significant decrease in the expression of *Cnr1*, encoding cannabinoid receptor 1 Type (CB1R), in the cerebral. This finding was consistently verified through western blot analysis, revealing a substantial reduction in CB1R content. Additionally, a significant decrease in the endocannabinoid 2-arachidonoylglycerol was observed in the cerebral cortex. Remarkably, administering the cannabinoid receptor agonist Win55-212-2 significantly alleviated the anxiety-like behavior, and the cannabinoid receptor antagonist AM251 effectively counteracted the anti-anxiety effects of Win55-212-2. In summary, our research confirmed that dual-frequency EMR is more likely to induce anxiety-like behavior in mice than single-frequency EMR, with implications for the hypothalamic–pituitary–adrenal axis and the endocannabinoid system. Furthermore, our findings suggest that Win55-212-2 may represent a novel avenue for researching and developing anti-EMR drugs.

## 1 Introduction

Recently, electromagnetic radiation (EMR) within the communication frequency band, include 2.65 and 0.8GHz, generated by wi-fi, mobile phones and mobile base stations, has become crucial component of environmental EMR. Concerns about its potential impact on human health, particularly in relation to brain function, causing sleep disorders (Danker-Hopfe et al., 2016) and increased reactive oxygen species (Choi et al., 2020), have spurred discussions in the field of electromagnetic biology (Kesari et al., 2013; Balmori, 2022; Bodewein et al., 2022).

Currently, there exists a disagreement regarding whether EMR exposure in the communication frequency band induces anxiety-like behavior, and different conditions may produce varying biological responses. For instance, mice exhibited anxiety-like behavior after 28 days of exposure to 2,450 MHz EMR (Gupta et al., 2018). In contrast, mice exposed to an 1800 MHz EMR from the global system for mobile phones for three days did not exhibit anxiety-like behavior (Júnior et al., 2014). Additionally, most previous research focused on electromagnetic waves with a single frequency, whereas real-life electromagnetic fields typically consist of multiple-frequency electromagnetic waves. Consequently, the differences between single- and multi-frequency EMR on emotional effects remain unknown.

The molecular mechanism through which EMR induces negative affects remains unclear. The hypothalamic–pituitary–adrenal (HPA) axis, a pivotal system during stress (Roozendaal et al., 2009), is often overactivated by long-term EMR exposure, acting as a stressor for mice (De Bruyn and De Jager, 1994). Exposure to 2.45 GHz EMR for two hours daily over 15, 30, and 60 days increased serum corticosterone (CORT) levels in mice, corresponding to the duration of exposure (Shahin et al., 2018). Prolonged EMR exposure activating the HPA axis is linked to the development of negative affect. Recent research highlights that the endocannabinoid system (ECS) is a fundamental regulator of HPA axis feedback inhibition and a crucial regulator of emotion (Finn, 2010; Hill et al., 2010). ECS, associated with various psychiatric diseases, especially anxiety-like behavior (Mangieri and Piomelli, 2007; Hill and Gorzalka, 2009), comprises cannabinoid receptors, endocannabinoids, and enzymes responsible for their synthesis and degradation. Cannabinoid receptor 1 type (CB1R) is abundant in the central nervous system, especially in the cortex and hippocampus (Mackie, 2005), and mice with full CB1R deletion exhibit more pronounced anxiety-like behavior (Jacob et al., 2009). Key endocannabinoids, including 2-arachidonoylglycerol (2-AG) and anandamide (AEA), can normalize anxiety-like behavior in mice by enhancing 2-AG signaling (Bosch-Bouju et al., 2016). Endocannabinoid degradation enzymes, such as fatty acid amide hydrolase (FAAH) and monoacylglycerol lipase (MGL), play crucial roles in ECS. FAAH is responsible for degrading AEA, and its gene deletion results in increased AEA content in the brain, leading to an improvement in anxiety-like behavior (Moreira et al., 2008). Conversely, MGL degrades 2-AG, and its overexpression reduces 2-AG levels, contributing to an increase in anxiety-like behavior (Guggenhuber et al., 2015). JZL184, an MGL inhibitor, crucially regulates MGL, inducing anxiolytic-like effects (Pavón et al., 2021). Thus, the ECS plays a vital role in regulating anxiety-like behavior and other negative effects caused by acute and chronic stress and repeated noise stimulation (Morena et al., 2016; Newsom et al., 2020). However, it remains unclear whether the ECS plays a role in the negative effects generated by EMR and can serve as a potential intervention target for EMR-induced brain injury.

Building upon the information provided, this study employed a reverberation chamber (RC) to establish three EMR animal models. These models were exposed to EMR of single frequencies, precisely 2.65 and 0.8 GHz, as well as a dual-frequency combination of 2.65/0.8 GHz. The objective was to investigate the emotional effects, neurobiological mechanisms, and potential intervention targets associated with long-term EMR exposure.

## 2 Results

### 2.1 Absence of anxiety-like behavior in mice exposed to single-frequency (2.65 or 0.8 GHz) electromagnetic radiation

**Figure 1:**
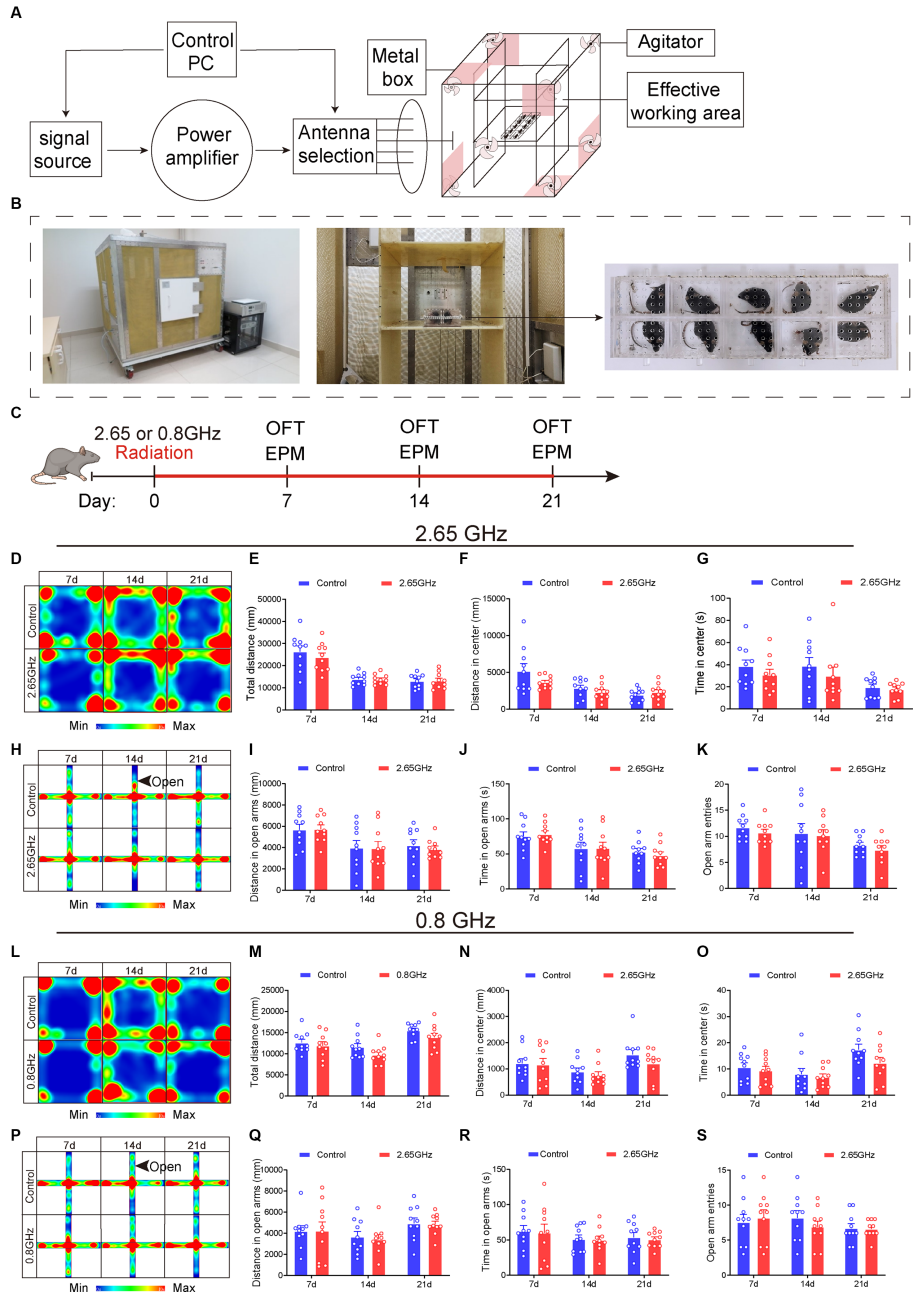
Electromagnetic radiation (EMR) exposure system and effects of single-frequency 2.65 or 0.8 GHz EMR on emotion. Schematic representation of RC modeling **(A)** and photos of the EMR exposure system **(B)**. **(C)** Experimental timeline for single-frequency 2.65 GHz radiation (8:00–12:00), open field (OFT, 18:00–20:00), or elevated plus maze (EPM, 20:00–22:00) tests. Experimental timeline for single-frequency 0.8 GHz radiation (8:00–12:00), open field (OFT, 8:00–10:00), or elevated plus maze (EPM, 10:00–12:00) tests. **(D)** Heatmap illustrating representative trajectories and statistical analysis of OFT results on days 7, 14, and 21 of single-frequency 2.65 GHz EMR (*n* = 10 per group). **(E)** Total distance. **(F)** Distance in the center. **(G)** Time in the center. **(H)** The heatmap displays representative trajectories and statistical analysis of EPM results on days 7, 14, and 21 of single-frequency 2.65 GHz EMR (*n* = 10 per group). **(I)** Distance in open arms. **(J)** Time in open arms. **(K)** Open arm entries. **(L)** The heatmap showcases representative trajectories and statistical analysis of OFT results on days 7, 14, and 21 of single-frequency 0.8 GHz EMR (*n* = 10 per group). **(M)** Total distance. **(N)** Distance in the center. **(O)** Time in the center. **(P)** The heatmap presents representative trajectories and statistical analysis of EPM results on days 7, 14, and 21 of single-frequency 0.8 GHz EMR (*n* = 10 per group). **(Q)** Distance in open arms. **(R)** Time in open arms. **(S)** Open arm entries. All data are expressed as means ± SEM. Unpaired *t*-test in **(E–G,I–O,Q–S)**.

The EMR exposure device employed in this investigation is illustrated in Figures 1A,B. Mice in the radiation group, situated within an animal radiation box (Bai li Acrylic Products Factory, Beijing, China), underwent irradiation within the effective work area of the RC. In contrast, the control group mice were also placed in a radiation box but were not subjected to irradiation to eliminate the influence of irrelevant variables.

To explore the frequency-dependent effects on mice behavior, single-frequency models of 2.65 or 0.8 GHz EMR with a specific absorption ratio (SAR) at 4 W/kg were established. The process of establishing these EMR models and conducting behavioral evaluations is depicted in Figure 1C. The exposure duration was 4 h per day for a total of 21 days, excluding days designated for behavioral testing (e.g., days 7, 14, and 21). The results demonstrated that, when compared with the control group, the single-frequency 2.65 or 0.8 GHz EMR groups exhibited no significant differences in center distance and center time in open field test (OFT) on day 7, 14 and 21 (*p* > 0.05, Figures 1D–G,L–O). Additionally, there were no significant differences in the distance time and entries into the open arm in the elevated plus maze (EPM) on day 7, 14, and 21 (*p* > 0.05, Figures 1H–K,P–S). These findings collectively indicate that prolonged exposure to single-frequency EMR (2.65 or 0.8 GHz) did not induce anxiety-like behavior in mice.

### 2.2 Induction of anxiety-like behavior and altered serum levels in mice exposed to dual-frequency (2.65/0.8 GHz) electromagnetic radiation

**Figure 2:**
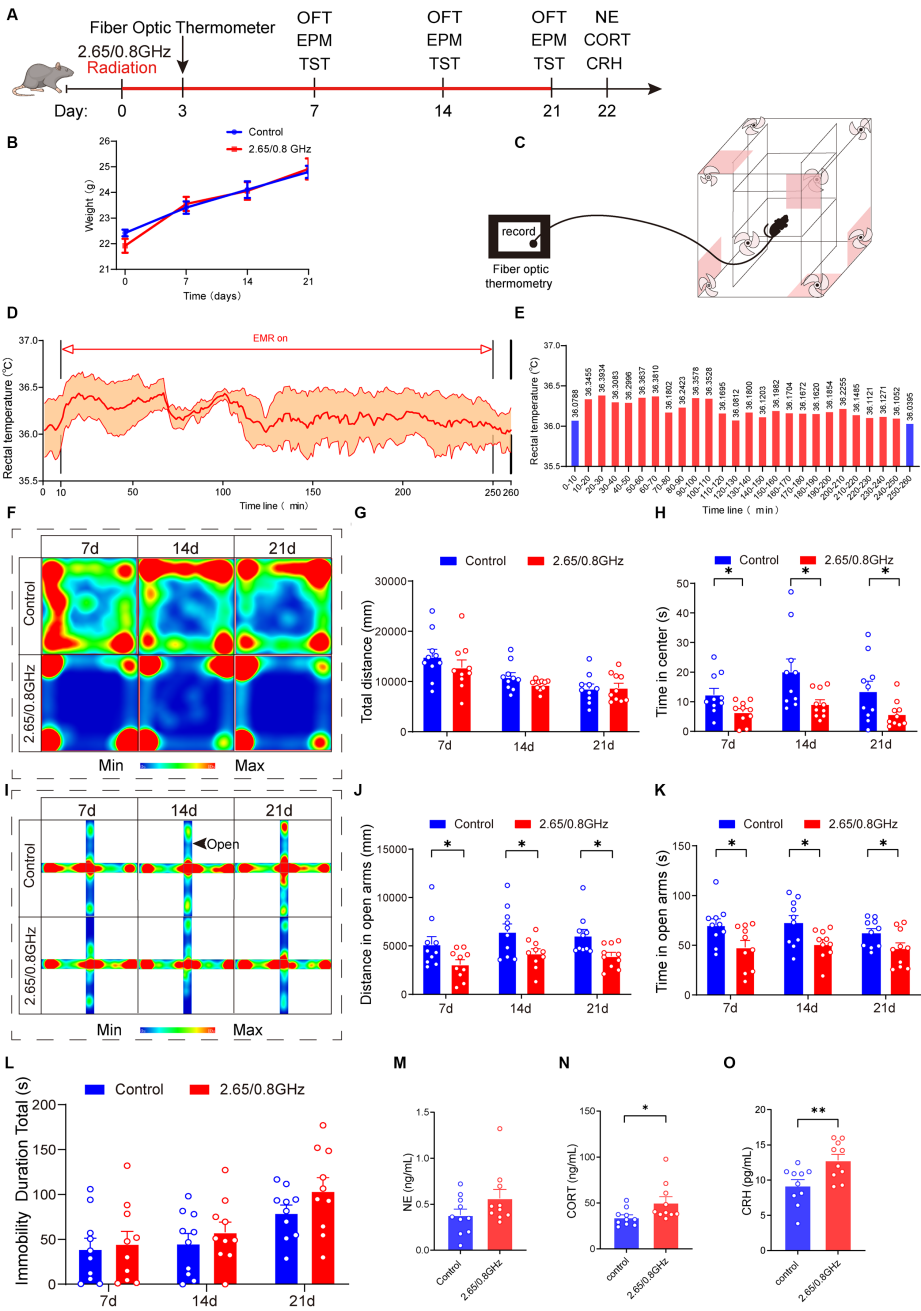
Effect of dual-frequency EMR on body weight, rectal temperature, emotion behavior, and serum hormones in mice. **(A)** Experimental timeline for dual-frequency 2.65/0.8GHz radiation (8:00–12:00), fiber optic thermometer, open field test (OFT, 8:00–10:00), elevated plus maze (EPM, 12:00–14:00), tail suspension test (TST, 18:00–20:00), and detection of NE, CORT, and CRH. **(B)** Changes in body weight of mice due to dual-frequency EMR (*n* = 10 per group). **(C–E)** Rectal temperature measured by a fiber optic thermometer (*n* = 3 per group). Schematic of the experimental device. **(D)** Plot of average rectal temperature before, during, and after EMR exposure. The red shadow indicates the standard error of the mean (SEM). **(E)** Histogram of rectal temperature as displayed in **(D)**. **(F)** Heatmap illustrating representative trajectories and statistical analysis of OFT results on days 7, 14, and 21 (*n* = 10 per group). **(G)** Total distance. **(H)** Time in the center. **(I)** Heat map showing representative trajectories and statistical analysis of EPM results on days 7, 14, and 21 (*n* = 10 per group). **(J)** Distance in open arms. **(K)** Time in open arms. **(L)** Statistical analysis of TST results on days 7, 14, and 21 (*n* = 10 per group). **(M)** There was no change in serum hormone NE content in mice (*n* = 10 per group). **(N)** Serum CORT increased significantly in mice (*n* = 10 per group). **(O)** Serum CRH increased significantly in mice (*n* = 10 per group). All data are expressed as means ± SEM. * *p* < 0.05, ** *p* < 0.01, control vs. 2.65/0.8GHz, repeated-measures analysis of variance in **(B)**, unpaired *t*-test in **(G,H,J–O)**.

To investigate the impact of multi-frequency EMR on brain function, we established a dual-frequency (2.65/0.8 GHz) EMR model, exploring both behavioral effects and molecular mechanisms (Figure 2A). Mice were randomly assigned to control and dual-frequency 2.65/0.8 GHz EMR groups. The dual-frequency group underwent exposure to 2.65 GHz radiation for 2 h, followed by 0.8 GHz radiation for an additional 2 h, totaling 4 h per day. The total radiation time (4 h) and SAR (4 W/kg) per day in the dual-frequency radiation group matched those in the single-frequency radiation group.

Mouse weights were measured during the radiation, revealing no differences between the control and dual-frequency (2.65/0.8 GHz) EMR groups (Figure 2B). Additionally, the rectal temperature of mice was monitored using a fiber-optic thermometer during dual-frequency (2.65/0.8 GHz) EMR exposure (Figure 2C), with ambient temperature at 18.6°C and humidity at 21.5%. The mean rectal temperature of mice before radiation was 36.0788°C, reaching a mean highest temperature of 36.3934°C and a mean lowest temperature of 36.0812°C (Figures 2D,E). This demonstrates that exposure to dual-frequency (2.65/0.8 GHz) EMR with SAR of 4 W/kg for 4 h caused a temperature increase of less than 1°C, allowing the research to focus on non-thermal effects while ruling out thermal effects in mice.

In the OFT (Figures 2F–H), the dual-frequency EMR group exhibited a significant reduction in center distance (Supplementary Figure 1A; *p* = 0.0217, day 7; *p* = 0.0094, day 14; *p* = 0.0382, day 21) and center time (Figure 2H; *p* = 0.0259, day 7; *p* = 0.0249, day 14; *p* = 0.0469, day 21) on day 7, 14, and 21 compared to the control group. In the EPM (Figures 2I–K; Supplementary Figure 1B), the dual-frequency EMR group displayed a significant reduction in distance (Figure 2J; *p* = 0.0402, day 7; *p* = 0.0293, day 14; *p* = 0.0111, day 21) and time (Figure 2K; *p* = 0.0307, day 7; *p* = 0.0231, day 14; *p* = 0.0448, day 21) in open arms on the same days. These findings indicate that dual-frequency (2.65/0.8 GHz) EMR induced anxiety-like behavior in mice. However, in comparison with the control group, the dual-frequency EMR group showed no significant difference in the total immobility duration during the tail suspension test (TST) on days 7, 14, and 21 (Figure 2L), suggesting that dual-frequency (2.65/0.8 GHz) EMR did not induce depression-like behavior in mice.

Furthermore, we analyzed serum hormones in mice, revealing no significant difference in norepinephrine (NE) content (*p* > 0.05, Figure 2M). However, noteworthy increases were observed in corticosterone (CORT) and corticotrophin releasing hormone (CRH) content (*p* = 0.0349, Figure 2N; *p* = 0.0078, Figure 2O) in the dual-frequency EMR group compared with the control group. These findings suggest that the anxiety-like behavior induced by dual-frequency EMR (2.65/0.8 GHz) may be related to alterations in CORT and CRH levels within the HPA axis.

In this part of the study, we established that prolonged exposure to dual-frequency 2.65/0.8 GHz EMR, below the thermal threshold (≤ 1°C), exerts a detrimental impact on both emotion and serum hormones in mice. This impact includes the induction of anxiety-like behavior without depression-like behavior, along with an upregulation of CORT and CRH content.

### 2.3 Dual-frequency (2.65/0.8 GHz) electromagnetic radiation significantly reduced the expression of *Cnr1* and the content of cannabinoid receptor 1 type in mice cerebral cortex

**Figure 3:**
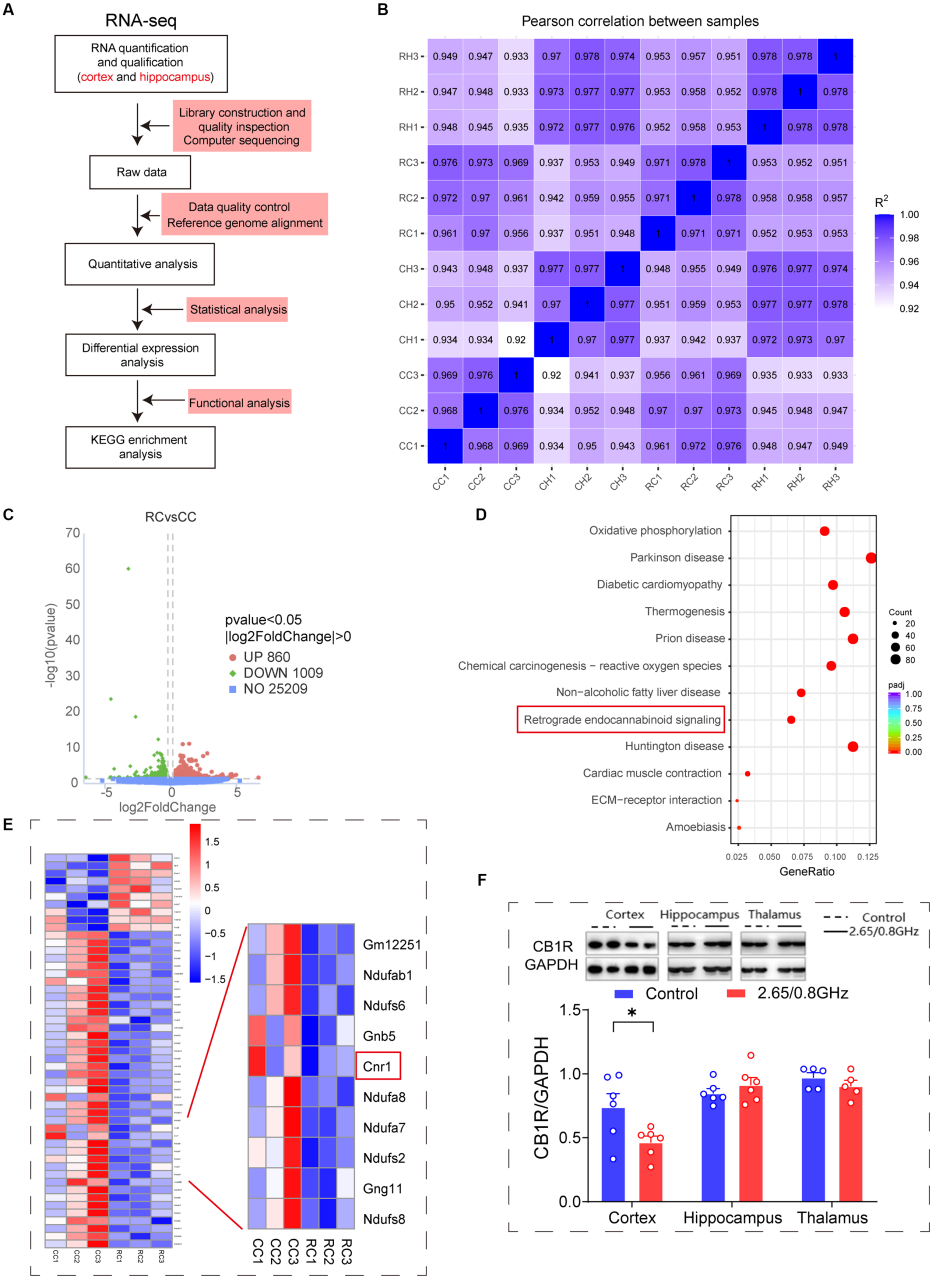
Impact of dual-frequency (2.65/0.8 GHz) EMR on *Cnr1* expression and CB1R protein levels in mouse brain cortex. **(A)** Workflow of transcriptome sequencing. **(B)** Sample correlation in mice cortical and hippocampal tissue. CC: Control+ cortex; CH: Control+ hippocampus; RC: Radiation+ cortex; RH: Radiation+ hippocampus. **(C)** Volcano plots illustrate the number of DEGs between the dual-frequency EMR group and the control group in the brain cortex (*p* < 0.05, *n* = 3 per group). **(D)** KEGG analysis of DEGs between the dual-frequency EMR group and the control group in the brain cortex. All signaling paths are listed, and with a focus on retrograde endocannabinoid signaling, w closely involved in mood regulation (*p* < 0.05, *n* = 3 per group). **(E)** Heat map displaying DEGs in retrograde endocannabinoid signaling. Several down-regulated genes are listed in the right panel, with a specific focus on the *Cnr1*, which encodes CB1R (*p* < 0.05, *n* = 3 per group). **(F)** Representative western blot and statistical analysis of CB1R/GAPDH in the brain cortex, hippocampus, and thalamus (*n* = 5–6 per group). Data are expressed as means ± SEM, *p < 0.05, unpaired *t*-test.

To investigate the molecular mechanisms triggering anxiety-like behaviors due to dual-frequency EMR, we extracted total RNA from the brain cortex and hippocampus of mice. We conducted an RNA-sequencing analysis (Figure 3A). The correlation of gene expression levels among all sequenced samples surpassed 0.92 (R^2^ > 0.92 represents ideal sampling and experimental conditions), indicating ideal sampling conditions and biological repeatability (Figure 3B).

In the brain cortex, 1869 differentially expressed genes (DEGs) were identified, consisting of 860 upregulated and 1,009 downregulated genes (Figure 3C). Kyoto encyclopedia of genes and genomes (KEGG) metabolic pathway analysis highlighted 12 significantly different metabolic pathways with a focus on the retrograde endocannabinoid signaling pathway (*p* = 6.41 × 10^−14^, consistent with the endocannabinoid system, ECS), closely linked to emotion regulation in mice (Figure 3D). Within this pathway, 51 DEGs were identified, including 10 upregulated and 41 downregulated DEGs. Compared to the control group, the *Cnr1* gene encoding CB1R was notably downregulated in the radiation group (*p* = 0.025, Figure 3E). Western blotting confirmed a significant reduction in CB1R protein expression in the cortex (*p* = 0.0367, Figure 3F), indicating that prolonged exposure to dual-frequency EMR impacted the CB1R expression of the ECS within the mice’s brain cortex. Similar analyses in the brain hippocampus revealed a significant difference in the retrograde endocannabinoid signaling pathway but no significant difference in *Cnr1* expression (Supplementary Figures S2A–C; *p* = 0.025, Supplementary Figure S2B). Western blotting for CB1R protein expression in the hippocampus and thalamus showed no difference in the dual-frequency EMR group compared to the control group (Figure 3F).

In summary, these results suggest that the downregulation of CB1R in the ECS within the brain cortex may lead to decreased binding with corresponding ligands, resulting in the downregulation of ECS function, ultimately leading to anxiety-like behavior in mice.

### 2.4 Dual-frequency (2.65/0.8 GHz) electromagnetic radiation significantly reduced the molecular levels of 2-AG in mice cerebral cortex

**Figure 4:**
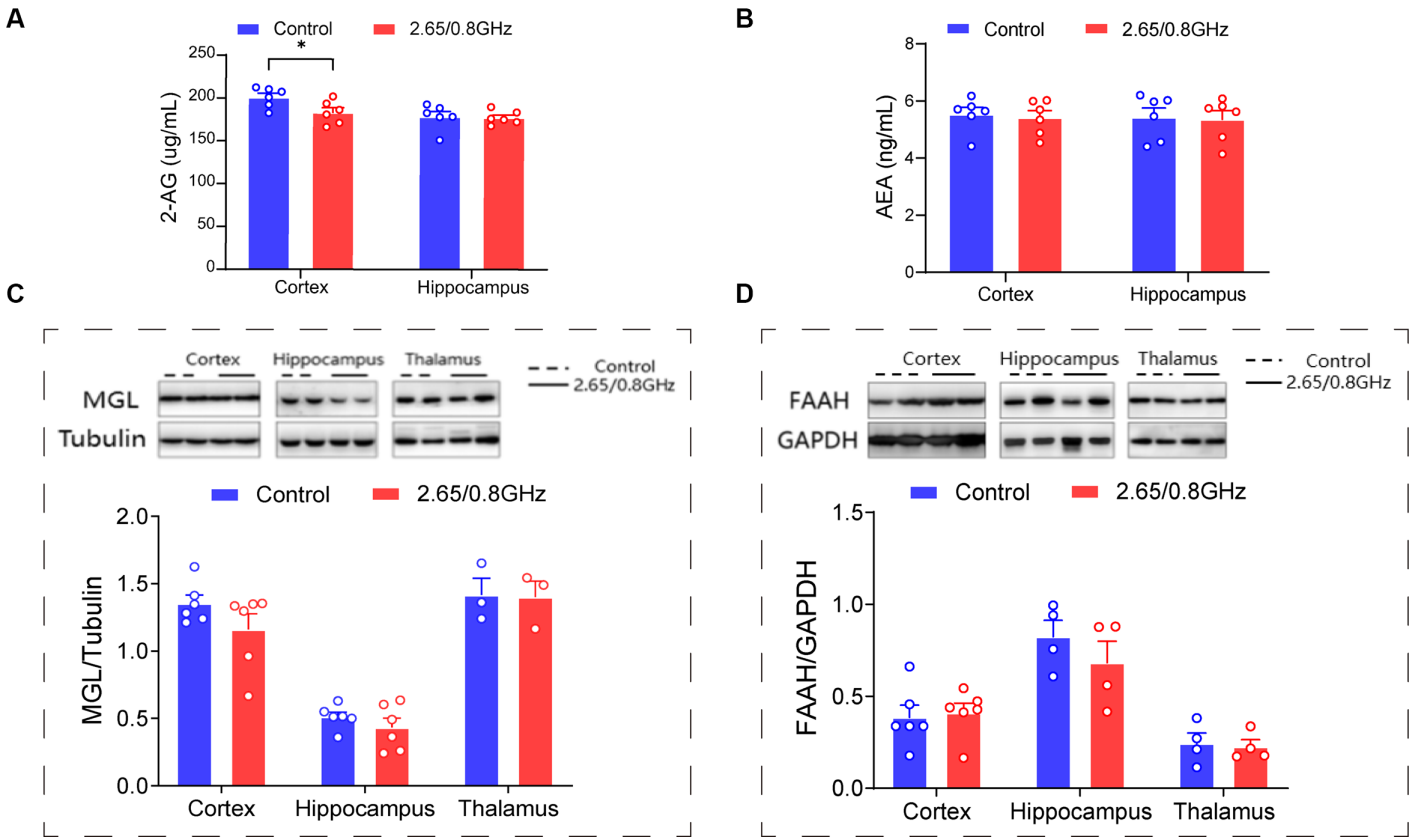
Impact of dual-frequency (2.65/0.8 GHz) EMR on 2-AG molecular levels in mouse brain cortex. **(A)** Changes in 2-AG molecular levels in the cortex and hippocampus (*n* = 6 per group). **(B)** Changes in AEA molecular levels in the cortex and hippocampus (*n* = 6 per group). **(C)** Representative western blot and statistical analysis of MGL/Tubulin in the cortex, hippocampus, and thalamus (*n* = 3–6 per group). **(D)** Representative western blot and statistical analysis of FAAH/GAPDH in the cortex, hippocampus, and thalamus (*n* = 4–6 per group). All data are expressed as means ± SEM, *p < 0.05, unpaired *t*-test in **(A–D)**.

Pertaining to the endocannabinoids 2-AG and AEA, crucial ligands of the ECS that predominantly bind to CB1R and regulate mood, we measured their molecular levels with an ELISA. Our analysis revealed a significant reduction in the levels of the 2-AG molecule in the cortex (*p* = 0.033, Figure 4A) and no significant change in the levels of the AEA molecule (*p* > 0.05, Figure 4B) in mice exposed to dual-frequency EMR compared to the control group. Furthermore, we investigated the enzymes responsible for controlling endocannabinoid levels in the brain, specifically MGL, which regulates 2-AG, and FAAH, which regulates AEA. However, our investigations did not unveil any significant alterations in the levels of MGL or FAAH enzymes (*p* > 0.05, Figures 4C,D).

These findings strongly suggest that exposure to dual-frequency EMR leads to the downregulation of the 2-AG ligand within the ECS of the brain cortex while leaving the levels of MGL and FAAH enzymes unaffected.

### 2.5 The cannabinoid receptor agonist Win55-212-2 improved anxiety-like behaviors, and restored serum hormone levels and endocannabinoid system expression induced by dual-frequency electromagnetic radiation

**Figure 5:**
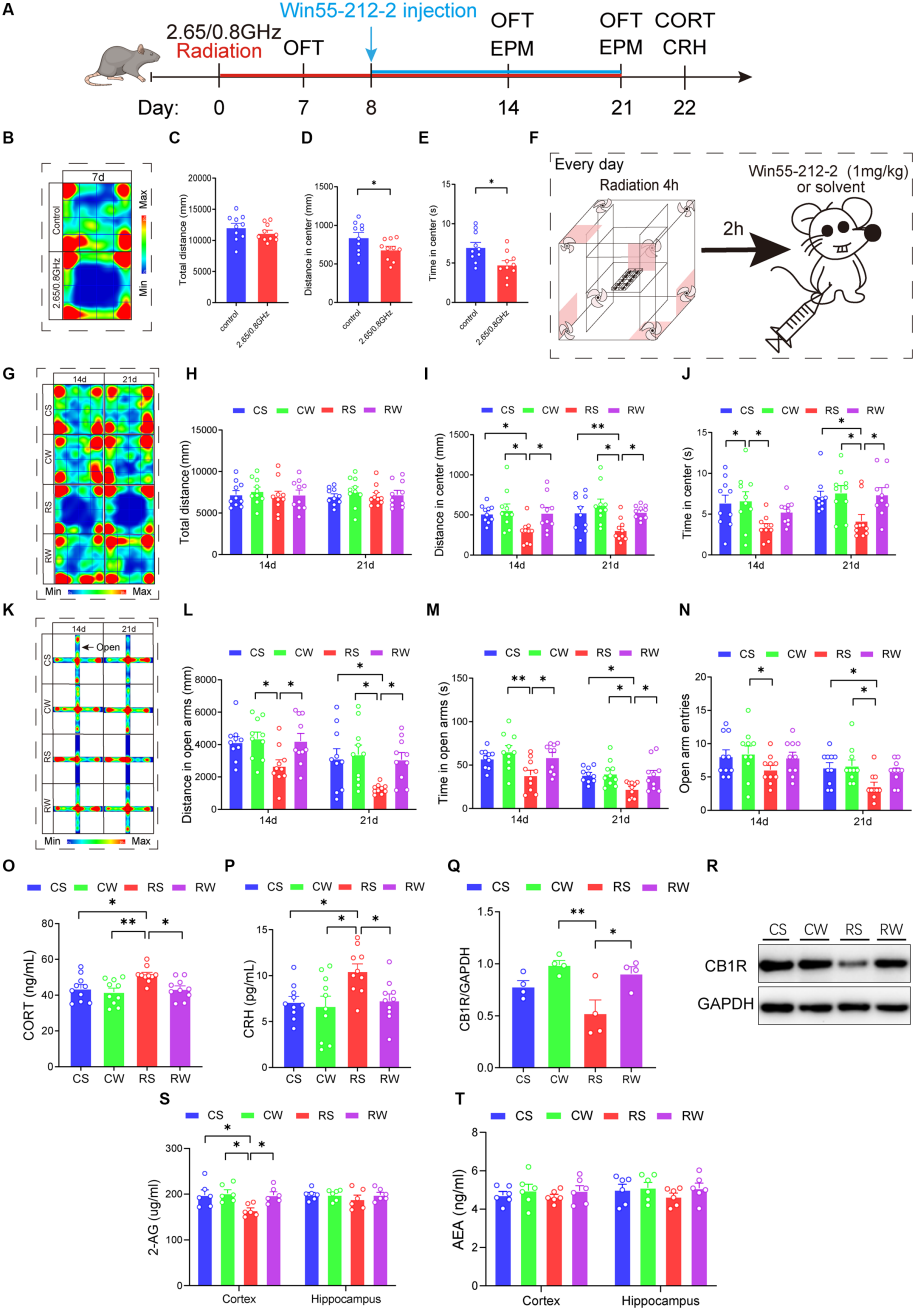
Win55-212-2, a cannabinoid receptor agonist, alleviates anxiety-like behavior and restores the content of serum hormone and endocannabinoid 2-AG levels. **(A)** Experimental timeline for 2.65/0.8 GHz radiation (8:00–12:00), open field (OFT, 8:00–10:00), elevated plus maze (EPM, 17:00–21:00), Win55-212-2 injection, and detection of CORT and CRH. Mice were radiated for a total of 21 days with the dual-frequency EMR and intraperitoneally injected with Win55-212-2 (1 mg/kg) or solvent from the 8th day to the 21st day. **(B)** Heatmap of representative trajectories and statistical analysis of OFT results on day 7 (*n* = 10 per group). **(C)** Total distance. **(D)** Distance in the center. **(E)** Time in the center. **(F)** Experimental timeline for Win55-212-2 daily injection. Mice were first irradiated for 4 h, with a 2 h interval, and then injected with Win55-212-2 or solvent daily from the 8th day to the 21st day. **(G)** Heatmap of representative trajectories and statistical analysis of OFT results on days 14 and 21 (*n* = 10 per group). **(H)** Total distance. **(I)** Distance in the center. **(J)** Time in the center. **(K)** Heatmap of representative trajectories and statistical analysis of EPM results on days 14 and 21 (*n* = 10 per group). **(L)** Distance in open arms. **(M)** Time in open arms. **(N)** Open arm entries. **(O)** Serum CORT content was significantly recovered in mice after the Win55-212-2 intervention (*n* = 10 per group). **(P)** Serum CRH content significantly recovered in mice after Win55-212-2 intervention (*n* = 10 per group). Statistical analysis **(Q)** and representative immunoblotting **(R)** of cortical CB1R/GAPDH after Win55-212-2 intervention (*n* = 4 per group). **(S)** Changes in 2-AG content in the cortex and hippocampus after Win55-212-2 intervention (*n* = 6 per group). **(T)** Changes in AEA content in the cortex and hippocampus after Win55-212-2 intervention (*n* = 6 per group). All data are expressed as means ± SEM, * *p* < 0.05, **p < 0.01, unpaired *t*-test in **(C–E)**, one-way ANOVA in **(H–J,L–T)**. CS: Control + solvent; CW: Control + Win55-212-2; RS: Radiation (2.65/0.8 GHz) + solvent; RW: Radiation (2.65/0.8 GHz) + Win55-212-2.

Given the pivotal role of the ECS in emotion regulation and the observed changes in ECS activity in the brains of mice exposed to dual-frequency EMR in this study, our next objective was to investigate whether increasing ECS activity could mitigate neurobehavioral harm. Starting from the 8th to 21st day post-EMR exposure, mice in the RW group received daily intraperitoneal injections of Win55-212-2, a cannabinoid receptor agonist obtained from Shanghai Yi Xi Chemical Technology Co., LTD, at a dose of 1 mg/kg. To provide context, this meant that mice weighing 20 g were injected with 0.02 mg of Win55-212-2. Mice in the CS and RS groups received an equivalent amount of solvent (a mixture of 10% Tween 80, 10% DMSO, and sterile saline) through intraperitoneal injections on a daily basis (Figure 5A).

During the initial week without Win55-212-2 or solvent treatment, radiation-exposed mice exhibited a significant reduction in center distance and center time in the OFT compared to the control mice (Figures 5B–E; *p* = 0.0337, Figure 5D; *p* = 0.0114, Figure 5E), indicating induced anxiety-like behavior. Following Win55-212-2 or solvent treatment Figure 5F, the results showed that, compared with the RS group, the RW group exhibited a significant increase in center distance in the OFT (Figures 5G–J; *p* = 0.0146, Figure 5I), and increased distance and time in open arms of the EPM on day 14 (Figures 5K–N; *p* = 0.045, Figure 5L; *p* = 0.045, Figure 5M). On day 21, compared with the RS group, the RW group displayed increased center distance and time in OFT (Figures 5G–J; *p* = 0.0406, Figure 5I); *p* = 0.0297, Figure 5J) and increased distance and time in open arms of EPM (Figures 5K–N; *p* = 0.0427, Figure 5L; *p* = 0.0443, Figure 5M). These results demonstrate that dual-frequency EMR caused anxiety-like behaviors in mice, and Win55-212-2 significantly alleviated these behaviors.

Moreover, we evaluated hormone levels in the serum, revealing that the RW group exhibited significantly lower CORT (*p* = 0.032, Figure 5O) and CRH contents (*p* = 0.0407, Figure 5P) compared to the RS group. Notably, no significant difference was observed in CORT and CRH content between the RW and CS groups. These findings imply that Win55-212-2 effectively reinstates serum CORT and CRH levels in response to dual-frequency EMR exposure. Subsequently, we assessed the CB1R content (Figures 5Q–R), uncovering that the RW group demonstrated a significant increase (*p* = 0.0237, Figure 5Q) compared to the RS group, with no notable difference between the RW and CS groups. These results signify that dual-frequency EMR induces downregulation of CB1R in the mouse cerebral cortex, which Win55-212-2 can efficiently ameliorate. In the final phase of our investigation into the effects of Win55-212-2 on endocannabinoid content in the brain cortex and hippocampus, we measured 2-AG and AEA contents (Figures 5S–T). The 2-AG content in the cortex of the RW group was significantly restored compared to the RS group (*p* = 0.0404, Figure 5S), indicative of Win55-212-2’s effectiveness in alleviating the downregulation of 2-AG induced by dual-frequency EMR.

In conclusion, Win55-212-2 may ultimately improve anxiety-like behavior induced by dual-frequency EMR by restoring HPA axis serum hormone levels and brain cortex 2-AG and CB1R content.

### 2.6 The role of cannabinoid receptor in anxiety and the antagonistic effect of AM251 on Win55-212-2

**Figure 6:**
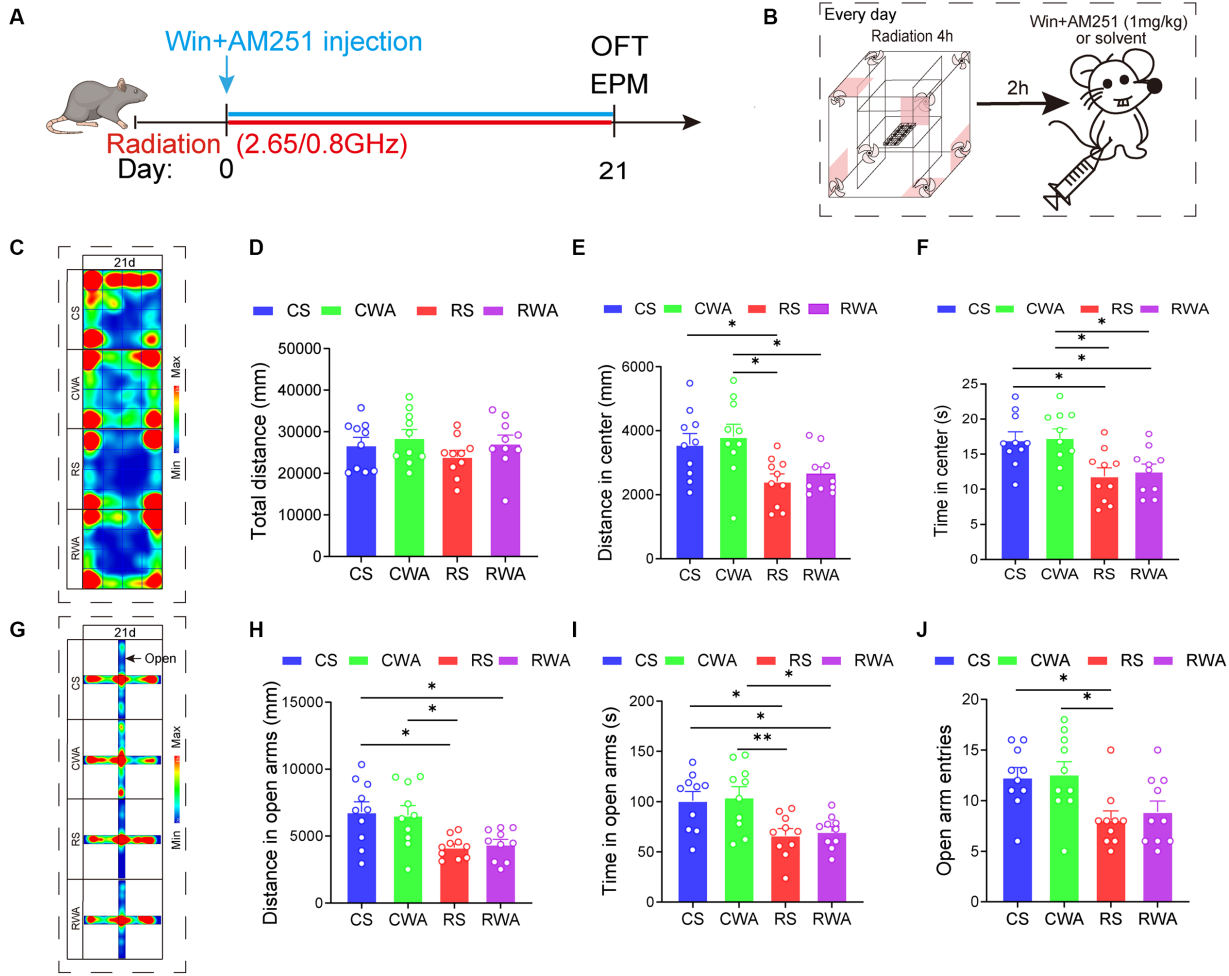
AM251, an endocannabinoid antagonist, antagonizes the anti-anxiety effects of Win55-221-2. **(A)** Experimental timeline for 2.65/0.8 GHz radiation (8:00–12:00), open field (OFT, 17:00–18:30), elevated plus maze (EPM, 18:30–22:00), WinAM251 injection. Mice were radiated for 21 days with dual-frequency EMR and intraperitoneally injected with WinAM251 (1 mg/kg) or solvent. **(B)** Experimental timeline for WinAM251 daily injection. Mice were first radiated for 4 h, with an interval of 2 h, and then injected with WinAM251 or solvent every day from the 1st day to the 21st day. **(C)** Heatmap of representative trajectories and statistical analysis of OFT results on day 21 (*n* = 10 per group). **(D)** Total distance. **(E)** Distance in the center. **(F)** Time in the center. **(G)** Heatmap of representative trajectories and statistical analysis of EPM results on day 21 (*n* = 10 per group). **(H)** Distance in open arms. **(I)** Time in open arms. **(J)** Open arm entries. All data are expressed as means ± SEM, *p < 0.05, **p < 0.01, one-way ANOVA in **(D–J)**. CS: Control + solvent; CWA: Control + WinAM251; RS: Radiation (2.65/0.8 GHz) + solvent; RWA: Radiation (2.65/0.8 GHz) + WinAM251.

Given that Win55-212-2 acts as an agonist for the cannabinoid receptors CB1 and CB2, we aimed to elucidate the specific involvement of CB1R in anxiety induced by dual-frequency EMR. To assess this, we employed the CB1R antagonist AM251 to ascertain whether the anti-anxiety effects of Win55-212-2 could be inhibited. The RWA group mice received intraperitoneal injections of WinAM251 (Win55-212-2 and AM251 combination, a cannabinoid receptor antagonist, APExBIO Technology LLC, USA) daily, with a dose of 1 mg/kg. In contrast, mice in the CS and RS groups were intraperitoneally injected with an equal amount of solvent (a mixture of 10% Tween 80, 10% DMSO, and sterile saline) daily (Figure 6A).

Upon completion of the WinAM251 or solvent treatment (Figure 6B), the results revealed that, in comparison with the RS group, there were no significant differences in the center distance and center time of the OFT (Figures 5C–F). Similarly, there were no significant differences in the distance and time in the open arms of the EPM (Figures 5G–J) in the RWA group on day 21. We already know that Win55-221-2 can ameliorate anxiety-like behavior induced by dual-frequency EMR. However, following the WinAM251 treatment, the anxiety-like behavior did not improve. These findings indicate that AM251 effectively antagonizes the anti-anxiety effects of Win55-221-2, suggesting a direct association between anxiety-like behaviors induced by dual-frequency EMR in mice and the CB1R of the ECS.

## 3 Discussion

In prior investigations, the impact of EMR on emotion predominantly relied on single-frequency EMR, with limited exploration into the neurobehavioral effects of dual-frequency EMR. In this study, we observed that under SAR of 4 W/kg, prolonged exposure to single-frequency 2.65 or 0.8 GHz EMR did not induce anxiety-like behavior in mice. Conversely, exposure to dual-frequency 2.65/0.8 GHz EMR, below the thermal threshold (≤ 1°C), triggered anxiety-like behavior without causing depression-like symptoms. This suggests that the frequency complexity of electromagnetic fields may be a key factor influencing negative effects. Serum CORT and CRH levels significantly increased, while the expression of Cnr1 and the content of CB1R and 2-AG in the cerebral cortex significantly decreased after long-term exposure to dual-frequency 2.65/0.8 GHz EMR. Additionally, the cannabinoid receptor agonist Win55-212-2 significantly improved the anxiety-like behavior, while the cannabinoid receptor antagonist AM251 effectively countered the anti-anxiety effects of Win55-221-2. Therefore, our findings suggest that the long-term impact of EMR on anxiety-like behavior is frequency-dependent, mediated by the ECS. These findings provide crucial insights into understanding the characteristics and mechanisms of 2.65/0.8 GHz EMR effects on the nervous system, offering potential intervention targets for protection against such EMR.

The RC is a rectangular resonator comprised of metallic conductors in an ideal state, possessing characteristics such as tunable frequency and a uniform electromagnetic field in its efficient working region. Mice in the radiation group were exposed to 4 h per day for a total of 21 days, simulating the situation that people are in the environment of communication frequency band EMR that people experience in their daily lives. Our study revealed that a single frequency of 2.65 or 0.8 GHz EMR did not cause anxiety-like behavior in mice, whereas dual-frequency 2.65/0.8 GHz EMR did. These findings suggest that the emotional effects of EMR may be sensitive to complex frequencies. Previous studies have demonstrated that 6 min exposure to combined electromagnetic fields of 1.5 GHz (SAR: 3.7 W/kg) and 4.3 GHz (SAR: 3.3 W/kg) resulted in more severe cognitive and memory damage compared to single-frequency groups (Zhu et al., 2021). This aligns with our study, indicating that EMR of complex frequency may enhance the damage to mood and mental status.

It has been reported that the EMR activates the HPA axis (Gupta et al., 2019) and its receptors (Hosseini et al., 2022), influencing neurotransmitter release by activating voltage-gated calcium channels and elevating intracellular calcium levels (Pall, 2016; Othman et al., 2017) in mice. In this study, we observed a significant increase in serum CORT and CRH levels in mice following long-term exposure to dual-frequency EMR, suggesting an association between anxiety-like behavior and activated HPA axis. The activated HPA axis, in turn, influences the ECS (Campolongo et al., 2011; Llorente-Berzal et al., 2011). For instance, CRH, by activating the CRH1 type receptor, leads to rapid FAAH production, driving AEA hydrolysis in the amygdala and ultimately leading to anxiety-like behavior (Gray et al., 2015). The ECS, known as a global regulator of stress responses (Bellocchio et al., 2013), regulates various brain functions by generating AEA and 2-AG. These endocannabinoids regulate synaptic strength through retroactive signals in conjunction with presynaptic CB1R (Castillo et al., 2012). Chronic stress can result in CB1R downregulation and alterations in AEA and 2-AG contents (Rademacher et al., 2008; Wamsteeker et al., 2010). The deficiency of CB1R, AEA, and 2-AG can induce anxiety-like behaviors while increasing AEA and 2-AG can have anti-anxiety effects. Previous studies have demonstrated that EMR affects the metabolism and transport of neurotransmitters, including biogenic amines such as 5-hydroxytryptamine, amino acids like γ-aminobutyric acid (GABA) and glycine, and peptide neurotransmitters such as endogenous opioid peptides (Hu et al., 2021). In our studies, we found that long-term dual-frequency EMR significantly reduced the expression of Cnr1 and the content of CB1R and 2-AG in the cerebral cortex of mice. This finding provides a plausible mechanism linking anxiety-like behaviors to alterations in the ECS.

Dual-frequency EMR may induce anxiety-like behavior by influencing the ECS in key brain regions. In comparison with the control group, the CB1R content in the hippocampus and thalamus remained unchanged in the radiation group, as did 2-AG in the hippocampus; significant changes were observed only in the cerebral cortex. Similar findings were noted in the cerebral cortex, where increased 2-AG levels of Fragile X Messenger Ribonucleoprotein mice correlated with improved anxiety-like behavior (Pirbhoy et al., 2021). This implies that anxiety-like behavior is region-specific and is primarily triggered by ECS dysregulation in the cerebral cortex, possibly due to increased energy absorption during EMR exposure compared to the hippocampus and thalamus (Cardis et al., 2008). Additionally, neural activity in the ventromedial prefrontal cortex (vmPFC) has been identified as a key factor in stress-induced anxiety-like behavior. Pharmacological activation of the vmPFC has been shown to effectively prevent this type of anxiety (Worley et al., 2020), with the ECS playing a pivotal role (Almeida-Santos et al., 2017) through AEA, 2-AG, CB1R and CB2R (McLaughlin et al., 2014). Consequently, future research should investigate the impact of dual-frequency EMR on the vmPFC. Moreover, we observed no significant difference in the expression levels of FAAH and MGL, the two major enzymes responsible for regulating endocannabinoid content to maintain ECS stability in the brain. This suggests that changes in endocannabinoid content in the cerebral cortex were unrelated to FAAH and MGL, warranting further studies on other enzymes.

The ECS’s role is to provide an intervention target for the biological effects of EMR in this research. However, prevention and protection against EMR are seldom reported. Win55-212-2 has been reported to improve anxiety-like behavior resulting from repeated social defeat and post-traumatic stress disorder in mice (Lisboa et al., 2018; Vimalanathan et al., 2020). As a cannabinoid receptor agonist, Win55-212-2 primarily improves anxiety-like behavior by modulating endocannabinoid binding to CB1R. Our findings indicate that Win55-212-2 significantly restored 2-AG and CB1R content in the cerebral cortex, improving anxiety-like behavior induced by long-term dual-frequency EMR.

Furthermore, it has been reported that intraperitoneal injection of the CB1R antagonist AM251 negatively affects the anti-anxiety action of transcranial direct current stimulation (Fang and Wang, 2021). Similarly, our study found that AM251 intraperitoneal injection effectively antagonized the anti-anxiety effects of Win55-221-2, indicating that anxiety induced by dual-frequency EMR can be well regulated by interfering with CB1R function in the ECS. Additionally, Win55-212-2 significantly restored serum CORT and CRH levels, indicating that the ECS’s role in anxiety-like behavior may depend on its interaction with the HPA axis (Gorzalka and Hill, 2009; Hill and Tasker, 2012). Further research is needed to elucidate the relationship between the ECS and HPA axis during anxiety-like behavior caused by EMR. In short, the use of Win55-212-2 and AM251 confirmed the involvement of the ECS and the HPA axis in the anxiety-like behavior induced by dual-frequency EMR. Moreover, as Win55-212-2 significantly improved anxiety-like behavior, it could provide a new strategy for the research and development of anti-EMR drugs.

Nevertheless, our study had some limitations. First, the frequency of EMR in the environment is complex and diverse, so dual-frequency EMR cannot completely simulate the EMR situation in the environment. Second, the relationship between the HPA axis and ECS in anxiety-like behavior induced by dual-frequency EMR remains unclear. Third, we have only conducted research on male mice, while the impact of EMR on female mice remains to be studied. More research is needed to gain a better understanding of these issues.

## 4 Materials and methods

### 4.1 Animals

Eight-week-old male C57BL/6 J mice (20.00 ± 0.43 g) were purchased from SPF Biotechnology Co., LTD. (Beijing, China). The mice were housed in standard laboratory conditions, maintaining a 12 h light–dark cycle (7:30 to 19:30) at an ambient temperature of 22 ± 2°C and a relative humidity of 50–60%. The mice *ad libitum* access to a standard diet and tap water freely. All experimental animal procedures were approved by the Institutional Animal Care and Use Committee of the National Beijing Center for Drug Safety Evaluation and Research.

### 4.2 EMR exposure equipment

The radiation equipment used was a general electromagnetic RC constructed by the Tongning Wu team of the Environmental and Security Department of the China Academy of Information and Communication Technology (Li et al., 2016). The RC basic equipment consists of a shielding body, radio frequency (RF) shield door, and RF agitator, and essential equipment includes the signal source and power amplifier. RC is frequently employed to simulate EMR environments and is widely used in electromagnetic biomedicine and conservation research. RC offers notable advantages. On the one hand, it provides effective shielding to prevent external electromagnetic environment interference, eliminating EMR contamination. On the other hand, the electromagnetic environment it produces has a uniform field strength, making it suitable for biological experiments.

RC supports radiative modeling at any frequency between 0.8 and 4 GHz. The experimental parameters are detailed in Table 1, with frequencies set at 2.65 and 0.8 GHz and the electric field intensity calculated based on the mouse’s body weight, ensuring a specific absorption ratio (SAR) of 4 W/kg. According to guidelines from the International Commission on Non-Ionizing Radiation Protection (ICNIRP, 2020), 4 W/kg is considered a safety threshold. This indicates the radiation intensity at which the body core temperature increases by 1°C after 30 min of whole-body exposure (ICNIRP, 2020). The radiation duration of mice was 4 h per day, from 8:00 to 12:00.

**Table 1:** Corresponding electric field strength parameters in mice with different body weights and frequencies.

Weight of mice (g)	Radiation frequency (GHz)	Electric field intensity (V/m)
21.9	2.65	188.93
25.0	2.65	189.79
30.0	2.65	190.00
21.9	0.80	243.00
25.0	0.80	262.00
30.0	0.80	285.00

### 4.3 Fiber optic thermometer

During radiation exposure, the rectal temperature of the mice was monitored in real-time using a fiber-optic thermometer (model THR-NC-1084C, FISO, China). The system includes an optical fiber temperature sensor, a rectal intervention catheter, and a mouse fixator. Mice were fixed within a conical cylinder mouse fixator made of acrylic material, with a tail baffle for stability to facilitate the measurement of mouse anus temperature. Placed on an RC-exposed platform, mice were lubricated with a small amount of Vaseline, and a metal-free fiber optic thermometer sensor (2 mm in diameter) was gently inserted into the anus to a depth of approximately 7 mm. The mouse’s anus temperature was measured in real-time, and rectal temperature data was recorded via the optical fiber before, during, and after radiation exposure.

### 4.4 OFT

The equipment was purchased from Shanghai Gilang Software Technology Co., LTD (Shanghai, China). OFT was performed in an open plastic box with a size of 50 × 50 × 40 cm^3^. Mouse trajectories were monitored for 5 min using the animal behavior analysis software. The total distance, center distance, and center time were used as analysis parameters to assess anxiety-like behavior. Prior to the experiment the mouse was placed in the experimental environment for more than 1 h to avoid stress. In addition, quiet was ensured during the trial. The apparatus was cleaned with a 75% ethanol solution before each mouse was introduced.

### 4.5 EPM

The equipment was purchased from Anhui Zhenghua Biological Instrument Equipment Co., LTD (Anhui, China). The EPM consisted of two open arms (40 × 5 × 10 cm^3^) and two closed arms (40 × 5 × 10 cm^3^) connected to a common central area (5 × 5 cm^2^). The apparatus was raised to a height of 50 cm from the ground. Mouse trajectories were monitored for 5 min using animal behavior analysis software. The distance and time in open arms, and open arm entries were used as parameters to assess anxiety-like behavior. Prior to the experiment the mouse was placed in the experimental environment for more than 1 h to avoid stress. In addition, quiet was ensured during the trial. The maze was cleaned with a 75% ethanol solution before each mouse was introduced.

### 4.6 TST

The tail suspension test is a classic experimental paradigm for evaluating depression-like behavior. Before initiating the experiment, the tape was wrapped approximately 1 cm from the tail tip of the mice. A clip was used to secure the tape, allowing the head of the mice to hand upside down on the support, with the nose tip kept approximately 15 cm away from the ground. Video equipment automatically recorded the activity of the mice for 6 min, with the first 2 min serving as an adaptation stage and the second 4 min being the test stage. The duration in which the mice gave up struggling and remained still within the 4-min period was statistically analyzed using animal behavior software to evaluate depression-like behavior. Additionally, a quiet environment was ensured during the trial, and the apparatus was cleaned with a 75% ethanol solution before introducing each mouse.

### 4.7 Serum hormone test

Following the behavioral experiments, mice were anesthetized by intraperitoneal injection of 1% sodium pentobarbital. After achieving complete anesthesia, blood was drawn from the tail vein of the mice. The collected blood was refrigerated at 4°C overnight, and the upper serum was obtained after centrifugation at 4000 rpm for 10 min. The NE and CRH contents were detected using the mouse NE/CRH ELISA kit (Shanghai Enzyme-Linked Biotechnology Co., LTD., China). In contrast, CORT content was seen utilizing the mouse CORT ELISA kit (Enzo, USA). The experimental procedures were conducted in accordance with the provided instructions.

### 4.8 RNA-sequencing

After achieving complete anesthesia, mice were dissected, and the brain cortex and hippocampus were extracted from three independent biological mice in each group for transcriptome analysis. RNA sequencing was performed by Novogene Co., LTD (Beijing, China). Genes with an adjusted *p*-value <0.05 were considered differentially expressed. A detailed protocol is provided in Supplementary Information.

### 4.9 Endocannabinoid test

The brains of six mice randomly sacrificed in each group were swiftly removed. Subsequently, the cerebral cortex and hippocampus of the mice were isolated and snap-frozen in liquid nitrogen, followed by storage at −80°C. The examination of endocannabinoids 2-AG and AEA in the cerebral cortex and hippocampus was conducted using the mouse endocannabinoids 2-AG/AEA ELISA kit (Shanghai FanTai Biotechnology Co., Ltd., China), and the experimental procedures were carried out according to the provided instructions.

### 4.10 Western blot analysis

The brains of six mice randomly sacrificed in each group were swiftly removed. Subsequently, we isolated the hippocampus, cortex, and thalamus of mice to be snap-frozen in liquid nitrogen and stored at −80°C. These brain tissues were ground at a ratio of 1:20, meaning 1 mg tissue was added to 20 μL RIPA lysate (Beijing Bomaide Biotechnology Co., Ltd., China), and the supernatant was obtained by centrifugation. The total protein concentration of the samples was then adjusted using the BCA kit (Thermo Fisher, USA), and 5 × SDS-PAGE loading buffer was added. The samples were boiled in a water bath at 100°C for 10 min, removed, cooled on ice, and stored at −20°C. Subsequently, gel (Beijing Pulilai Gene Technology Co., Ltd., China), sample loading (2 μg/μL, 6 μL), electrophoresis run (90 V, 20 min; 110 V, one hour), wet membrane transfer (400 mA, 90 min), and PVDF membrane wash in TBST for 5 min were carried out. The PVDF membrane was blocked in 5% skim milk powder for one hour, followed by incubation in the primary antibody (internal reference: GAPDH and TUBULIN; primary antibodies: CB1R, FAAH, and MGL, all at a ratio of 1:1000, Abcam, American) at 4°C overnight. The next day, after one hour of incubation at room temperature, the PVDF membranes were washed with TBST three times, each time for 10 min. Secondary antibodies (Goat anti-rabbit and goat anti-mouse, Beijing Rambolide Trading Co., LTD, China.) were incubated for one hour and washed three times in TBST for 15 min each time. The developer solution (Lablead, China) was prepared with a ratio of liquid A to liquid B of 1:1, and the exposure was conducted using a developer (Tanon, China). Image J- win64 1.51 software was used to quantify the grayscale values of the WB strips.

### 4.11 Statistical analysis

Statistical analysis was performed using GraphPad Prism 8.4.2 software, and the data are expressed as the mean ± standard error of the mean (M ± SEM). Differences between two groups were analyzed by unpaired *t*-test, and differences between multiple groups were tested by one-way ANOVA. *p*-values <0.05 were considered to indicate significant differences.

## 5 Conclusion

In conclusion, this study demonstrated that compared with single-frequency EMR, long-term dual-frequency EMR is more likely to induce anxiety-like behavior in mice. Furthermore, anxiety-like behavior caused by dual-frequency EMR is related to the HPA axis and ECS and primarily localized in the cerebral cortex, suggesting that CB1R of ECS could be a potential therapeutic target. Finally, we established that the cannabinoid receptor agonist Win55-212-2 can significantly alleviate anxiety-like behavior, presenting a potential new strategy for the research and development of anti-EMR drugs.

## Data availability statement

The RNA-seq data presented in the study are deposited in the SRA repository, accession number PRJNA1091226.

## Ethics statement

The animal study was approved by Animal Care and Use Committee of the National Beijing Center for Drug Safety Evaluation and Research. The study was conducted in accordance with the local legislation and institutional requirements.

## Author contributions

TX: Data curation, Formal analysis, Investigation, Methodology, Software, Writing – original draft, Writing – review & editing. R-HM: Data curation, Investigation, Methodology, Writing – original draft. CX: Formal analysis, Investigation, Resources, Supervision, Validation, Writing – review & editing. BS: Data curation, Methodology, Project administration, Software, Validation, Writing – review & editing. D-FY: Data curation, Formal analysis, Software, Validation, Visualization, Writing – review & editing. X-ML: Formal analysis, Investigation, Methodology, Software, Supervision, Writing – review & editing. DG: Investigation, Methodology, Software, Supervision, Validation, Writing – review & editing. Z-HL: Formal analysis, Methodology, Supervision, Validation, Visualization, Writing – review & editing. YG: Funding acquisition, Investigation, Resources, Supervision, Validation, Writing – review & editing. C-ZW: Investigation, Methodology, Resources, Supervision, Validation, Writing – review & editing.
