# Supplementary material for: The endocannabinoid system is involved in the anxiety-like behavior induced by dual-frequency 2.65/0.8 GHz electromagnetic radiation in mice
Source: Front Mol Neurosci. 2024 Apr 15;17:1366855. doi: 10.3389/fnmol.2024.1366855 (PMC11057378; doi:10.3389/fnmol.2024.1366855)
Supplement: Supplementary file 1 [file Data_Sheet_1.pdf]

Supplementary Material

1 Supplementary Materials and Methods

RNA sequencing libraries construction and data analysis

1.1 RNA quantification and qualification

(1) RNA purity was checked using the NanoPhotometer® spectrophotometer (IMPLEN, CA, USA).
(2) RNA integrity was assessed using the RNA Nano 6000 Assay Kit of the Bioanalyzer 2100 system (Agilent Technologies, CA, USA).

1.2 Library preparation for Transcriptome sequencing

A total amount of 1μg RNA per sample was used as input material for the RNA sample preparations. Sequencing libraries were generated using NEBNext® UltraTM RNA Library Prep Kit for Illumina® (NEB, USA) following manufacturer's recommendations and index codes were added to attribute sequences to each sample.

Briefly, mRNA was purified from total RNA using poly-T oligo-attached magnetic beads. Fragmentation was carried out using divalent cations under elevated temperature in NEBNext First Strand Synthesis Reaction Buffer(5X). First strand cDNA was synthesized using random hexamer primer and M-MuLV Reverse Transcriptase (RNase H-). Second strand cDNA synthesis was subsequently performed using DNA Polymerase I and RNase H. Remaining overhangs were converted into blunt ends via exonuclease/polymerase activities. After adenylation of 3' ends of DNA fragments, NEBNext Adaptor with hairpin loop structure were ligated to prepare for hybridization. In order to select cDNA fragments of preferentially 250~300 bp in length, the library fragments were purified with AMPure XP system (Beckman Coulter, Beverly, USA). Then 3 μl USER Enzyme (NEB, USA) was used with size-selected, adaptor-ligated cDNA at 37°C for 15 min followed by 5 min at 95 °C before PCR. Then PCR was performed with Phusion High-Fidelity DNA polymerase, Universal PCR primers and Index (X) Primer. At last, PCR products were purified (AMPure XP system) and library quality was assessed on the Agilent Bioanalyzer 2100 system.

1.3 Clustering and sequencing (Novogene Experimental Department)

The clustering of the index-coded samples was performed on a cBot Cluster Generation System using TruSeq PE Cluster Kit v3-cBot-HS (Illumia) according to the manufacturer's instructions. After cluster generation, the library preparations were sequenced on an Illumina Novaseq platform and 150 bp paired-end reads were generated.

1.4 Data Analysis

(1) Quality control

Raw data (raw reads) of fastq format were firstly processed through in-house perl scripts. In this step, clean data (clean reads) were obtained by removing reads containing adapter, reads containing ploy-N and low quality reads from raw data. At the same time, Q20, Q30 and GC content the clean data were calculated. All the downstream analyses were based on the clean data with high quality.

(2) Reads mapping to the reference genome

Reference genome and gene model annotation files were downloaded from genome website directly. Index of the reference genome was built using Hisat2 v2.0.5 and paired-end clean reads were aligned to the reference genome using Hisat2 v2.0.5. We selected Hisat2 as the mapping tool for that Hisat2 can generate a database of splice junctions based on the gene model annotation file and thus a better mapping result than other non-splice mapping tools.

(3) Quantification of gene expression level

FeatureCounts v1.5.0-p3 was used to count the reads numbers mapped to each gene. And then FPKM of each gene was calculated based on the length of the gene and reads count mapped to this gene. FPKM, expected number of Fragments Per Kilobase of transcript sequence per Millions base pairs sequenced, considers the effect of sequencing depth and gene length for the reads count at the same time, and is currently the most commonly used method for estimating gene expression levels.

(4) Differential expression analysis

(For DESeq2 with biological replicates) Differential expression analysis of two conditions/groups (two biological replicates per condition) was performed using the DESeq2 R package (1.16.1). DESeq2 provide statistical routines for determining differential expression in digital gene expression data using a model based on the negative binomial distribution. The resulting P-values were adjusted using the Benjamini and Hochberg's approach for controlling the false discovery rate Genes with an adjusted P-value <0.05 found by DESeq2 were assigned as differentially expressed.

(For edgeR without biological replicates) Prior to differential gene expression analysis, for each sequenced library, the read counts were adjusted by edgeR program package through one scaling normalized factor. Differential expression analysis of two conditions was performed using the edgeR R package (3.18.1). The P values were adjusted using the Benjamini & Hochberg method. Corrected P-value of 0.05 and absolute foldchange of 2 were set as the threshold for significantly differential expression.

(5) Enrichment analysis of differentially expressed genes

KEGG is a database resource for understanding high-level functions and utilities of the biological system, such as the cell, the organism and the ecosystem, from molecular-level information, especially large-scale molecular datasets generated by genome sequencing and other high-through put experimental technologies (http://www.genome.jp/kegg/). We used clusterProfiler R package to test the statistical enrichment of differential expression genes in KEGG pathways.

2 Supplementary Figures

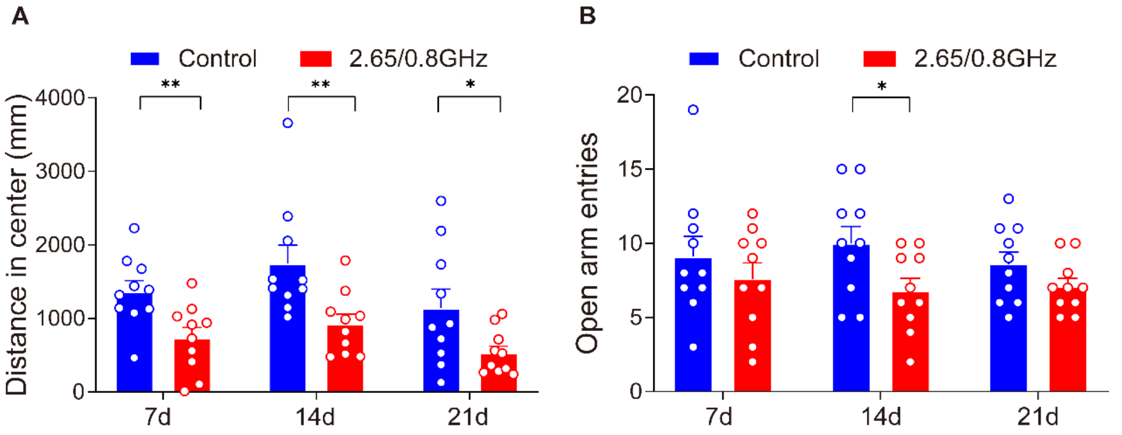

Supplementary Figure 1. Effects of dual-frequency EMR on mice anxiety-like behavior.

(A) Statistical analysis of distance in center of OFT results at day 7, 14, and 21(n = 10 per group). **(B)** Statistical analysis of open arm entries of EPM results at day 7, 14, and 21(n = 10 per group). All data are expressed as means ± SEM. * $p < 0.05$, ** $p < 0.01$, Control vs 2.65/0.8GHz, unpaired t-test in **A, B**.

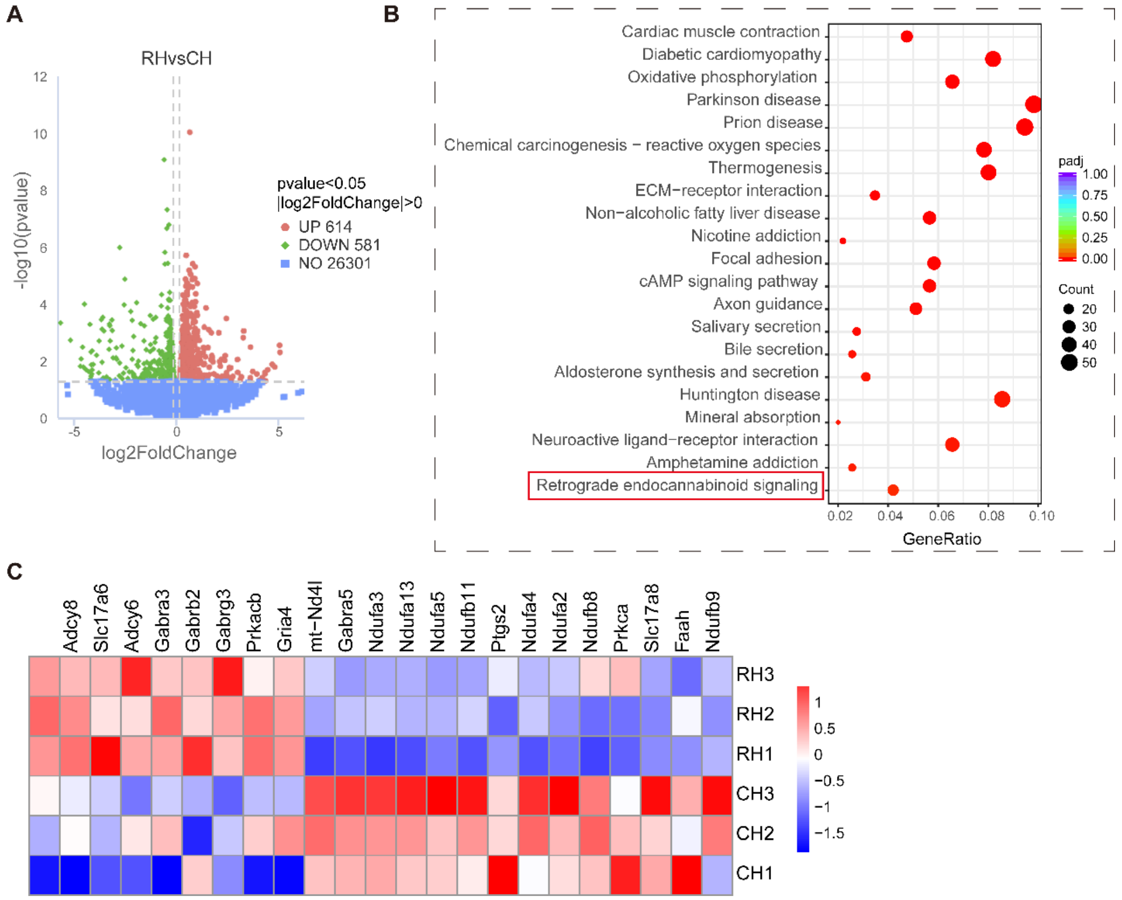

Supplementary Figure 2. Functional analysis based on RNA-seq data in the mouse brain hippocampus.

(A) Volcano plots showing the number of differentially expressed genes between dual-frequency EMR group and the control group in the hippocampus ($p < 0.05$, $n = 3$ per group). **(B)** KEGG analysis of differentially expressed genes between dual-frequency EMR group and the control group in the hippocampus. All signaling paths are listed, and we focused on the retrograde endocannabinoid signaling （$p = 0.0009$, p value < 0.05 were defined as significant）, which is closely involved in mood regulation ($n = 3$ per group). **(C)** Heat map showing differentially expressed genes in the retrograde endocannabinoid signaling. ($p < 0.05$, $n = 3$ per group). CC: Control+ cortex; CH: Control+ hippocampus; RC: Radiation+ cortex; RH: Radiation+ hippocampus.
